# Maternal, fetal and neonatal mortality: lessons learned from historical changes in high income countries and their potential application to low-income countries

**DOI:** 10.1186/s40748-014-0004-z

**Published:** 2015-01-22

**Authors:** Robert L Goldenberg, Elizabeth M McClure

**Affiliations:** Department of Obstetrics and Gynecology, Columbia University Medicine Center, New York, NY USA; Social, Statistical and Environmental Health Sciences, RTI International, Durham, NC USA

**Keywords:** Maternal mortality, Stillbirth, Neonatal mortality, Low-income countries

## Abstract

**Background:**

There are large differences in pregnancy outcome between high income countries and many middle and low income countries. In fact, maternal, fetal and neonatal mortality rates in many low-income countries approximate those that were seen in high-income countries nearly a century ago.

**Findings:**

This paper documents the very substantial reductions in maternal, fetal and neonatal mortality rates in high income countries over the last century and explores the likely reasons for those reductions. The conditions responsible for the current high mortality rates in low and middle income countries are discussed as are the interventions likely to result in substantial reductions in maternal, fetal and neonatal mortality from those conditions. The conditions that result in maternal mortality are often responsible for fetal and neonatal mortality and the interventions that save maternal lives often reduce fetal and neonatal mortality as well. Single interventions rarely achieve substantial reductions in mortality. Instead, upgrading the system of care so that appropriate interventions could be applied at appropriate times is most likely to achieve the desired reductions in maternal, fetal and neonatal mortality.

## Introduction

In many low-income countries, the maternal mortality ratios are 100-fold greater than in high-income countries (HIC) [1]. Rates of fetal and neonatal mortality are often 10-fold greater or more [2-5]. Current low-income country (LIC) maternal, fetal and neonatal mortality rates are generally similar to the HIC rates eighty to one hundred years ago [6,7]. This paper explores reasons that the rates of each of these outcomes have fallen in HIC and reviews the medical causes of maternal and neonatal mortality and stillbirth in LIC. Finally, we review current recommendations for interventions to reduce these mortality rates in LIC.

## Findings

### Maternal mortality

In most LIC, the major medical causes of maternal mortality, defined as deaths to the mother during pregnancy or in the first 42 days after birth, are hemorrhage, hypertensive diseases of pregnancy and various types of maternal infections [6,8]. Hemorrhage is often classified according to timing, i.e., occurring in the antepartum/intrapartum or during the post-partum period. The most common antenatal/intrapartum cause is placental abruption, while the most common cause of post-partum hemorrhage is failure of the uterus to contract after delivery or uterine atony [8,9]. The most dangerous hypertensive disease of pregnancy is preeclampsia, which may lead to a number of potentially deadly complications including strokes and seizures (eclampsia) [10]. Maternal infection includes bacterial sepsis, HIV, malaria, syphilis, and various other infections. Additionally, two other obstetric conditions are related to maternal mortality from hemorrhage and infection. Obstructed or prolonged labor may lead to maternal death from hemorrhage and infection - sometimes after uterine rupture. Women who undergo unsafe abortion may also die from complications of hemorrhage and infection.

### Stillbirth

Stillbirth, variably defined as deaths in utero from after 20 to 28 weeks gestation - depending on local standards, often results from maternal complications [11-13]. The most common maternal conditions leading to stillbirth include hypertensive diseases of pregnancy, especially preeclampsia and eclampsia, placental abruption, and various types of maternal infection, such as syphilis and malaria. Thus, most stillbirths result from asphyxia prior to or during labor, often associated with hypertensive diseases, abruption, obstructed or prolonged labor, and obstetric complications such as breech presentation and umbilical cord accidents. Treatment of maternal conditions, e.g., early delivery for preeclampsia, may reduce the associated fetal mortality but increase the risk of prematurity. Placental lesions, especially necrosis and thrombosis, but also placental signs of hemorrhage, are often discovered on placental histological examination in cases of stillbirth and some authors attribute the majority of fetal deaths to a placental abnormality [14,15].

### Neonatal mortality

Neonatal mortality, defined as death to a live-born baby within 28 days of life, in LIC is typically ascribed to three major causes: infection, asphyxia and prematurity [16]. The most common type of infections causing mortality are bacterial such as group B streptococcus (GBS), often acquired during labor, but infections causing mortality also may include malaria, syphilis, and tetanus [17,18]. Neonatal asphyxia is predominantly caused by maternal complications such as abruption, or preeclampsia [19]. Among preterm infants, conditions that contribute to mortality include 1) respiratory distress syndrome (RDS), 2) intraventricular hemorrhage (IVH), 3) necrotizing enterocolitis, and 4) infections [7,20,21]. In summary, a majority of all neonatal deaths are associated with one of the maternal or fetal conditions described above.

### History of pregnancy outcome improvements in high income countries and evidence-based interventions for low-income countries

#### Maternal mortality: history

**Figure 1:**
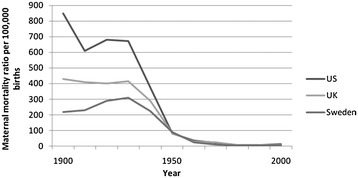
**Maternal mortality, 1900–2000, in select high-income countries.** Adapted from Goldenberg [6].

Significant reductions in maternal, fetal and neonatal mortality in HIC and many MIC’s have occurred during the last century. In these countries, until about 1935, maternal mortality ratios ranged from 500 to 1000 deaths per 100,000 births, i.e., nearly 1% of pregnant women died (Figure 1) [6,22-24]. Until about 1935, this situation in many countries had remained essentially unchanged for hundreds of years, although slow reductions in maternal mortality in Sweden have been attributed to the introduction of trained midwives [22]. In recent years, many HIC have reported maternal mortality ratios of about 10/100,000 births or less [1].

**Figure 2:**
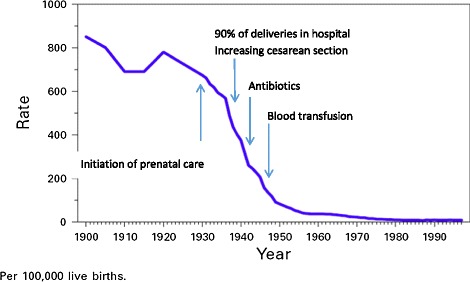
**Interventions associated with historical reduction in maternal mortality, United States, 1900–2000.** Adapted from Johnson 2001 [23].

Several papers have explored this reduction from 1935 through 1970–1980 [6,22-24]. Interventions responsible for the reductions included the introduction of prenatal care and hospitalization for delivery (1920’s and 1930’s), antibiotics to treat infection (late 1930’s and 1940’s), and uterotonics and blood transfusion (1940’s) (Figure 2). The management of preeclampsia/eclampsia steadily improved during the 1940’s to 1950’s with prenatal care to diagnose the condition (blood pressure measurements and urine protein determination), hospitalization to monitor the condition, and a transition from watchful waiting to immediate delivery for severe or progressing disease. With antibiotics, blood availability, and improvements in anesthesia, cesarean sections became safer and were more commonly used to terminate life-threatening pregnancies, such as those with prolonged labor or eclampsia. Thus, beginning around 1935, introduction of new, effective interventions were associated with significant decreases in maternal mortality. As these interventions were implemented, first in high-income and then in some middle-income countries, nearly a 99% reduction in maternal mortality was observed in these settings.

#### Maternal mortality: evidence-based recommendations

Prevention of hemorrhage, the leading cause of maternal mortality, can be accomplished through cesarean section for prolonged labor and the reduction of unsafe abortions [25,26]. For abortion-related complications, uterine suction or curettage, antibiotics, blood and sometimes hysterectomy prevent maternal mortality [26]. Use of uterotonics, such as misoprostol or oxytocin at delivery, can reduce post-partum hemorrhage associated with an atonic uterus [27]. The case fatality rate associated with hemorrhage may also be reduced through a reduction in anemia, usually accomplished prenatally with iron and vitamin supplementation [28]. However, major reductions in mortality from hemorrhage usually occur with appropriate treatment of hemorrhage once it occurs. Treatment varies with the cause of the hemorrhage and may include surgery for lacerations or a ruptured uterus, manual removal, suction or curettage for a retained placenta or placental fragments, uterotonics to treat hemorrhage from an atonic uterus, and for antepartum hemorrhage due to a placental abruption or previa, a cesarean section [9]. Hysterectomy (and sometimes other abdominal procedures) is the surgery of last resort for many types of obstetric hemorrhage. Also for hemorrhage of any type, blood products are often life-saving. An important message is that even within a single condition such as hemorrhage, there are many causes requiring substantial provider skills to assess the appropriate prevention and treatment. No single intervention is likely to have a large impact on hemorrhage-related maternal mortality.

After hemorrhage, the most frequent cause of maternal mortality is hypertensive disease, and particularly preeclampsia and eclampsia [10]. Prevention may be possible through maternal treatment with calcium and aspirin, but effectiveness of these interventions remains uncertain. Women die from many complications of preeclampsia/eclampsia including asphyxia during seizures, aspiration pneumonia, strokes and cardiac, liver and kidney failure, as well as hemorrhage secondary to clotting disorders and placental abruption [29]. Since preeclampsia is often asymptomatic until late in its course, diagnosing this condition early is the key to saving maternal lives. Since the condition is generally defined by hypertension and proteinuria, tests for both, repeated in prenatal care throughout the second half of pregnancy are required to effectively diagnose this condition. Once preeclampsia is diagnosed, close observation with delivery by induction of labor or cesarean section for worsening disease is often life-saving. Magnesium sulfate reduces initial and repeat seizures and may provide time to affect delivery prior to new or recurrent seizures [30]. Thus, a combination of prenatal care to diagnose the condition and facility care for delivery with labor induction and cesarean section capabilities are necessary to reduce maternal mortality from this condition.

Infection is the third major killer of mothers [31-33]. Malaria is an important cause of maternal death in endemic areas, and can be responsible for maternal deaths both during acute malarial episodes and after the acute episode due to its effect on maternal anemia [31]. Most important, however, are the bacterial infections of the uterus [32]. During and immediately after the delivery, the uterus provides an excellent culture medium for bacteria, and uterine infections are very common. The use of non-sterile delivery techniques, prolonged labor, and instrumentation including cesarean section all increase the risk of infection [34]. Prevention of infection includes use of clean delivery sites, provider hand washing, and avoiding prolonged labors and instrumentation whenever possible. Treatment generally consists of the timely administration of appropriate antibiotics.

In summary, most maternal deaths can be prevented by decreasing the prevalence of the conditions that cause the deaths or providing appropriate treatment for those conditions. Since, with a few important exceptions, most complications leading to maternal death cannot be predicted or prevented, care for these conditions must be readily available to all women. Most maternal deaths occur during labor, delivery and in the immediate post-partum period [6,24]. When the conditions causing maternal death are considered, it becomes clear that the treatments for these conditions need to be readily available during labor and after delivery. Few of these interventions are available in the home and unfortunately, in most LICs, many of these interventions are not available in the clinics and even in some hospitals. For these reasons, many have advocated for hospital deliveries, with these interventions available [35,36].

#### Fetal mortality: history

**Figure 3:**
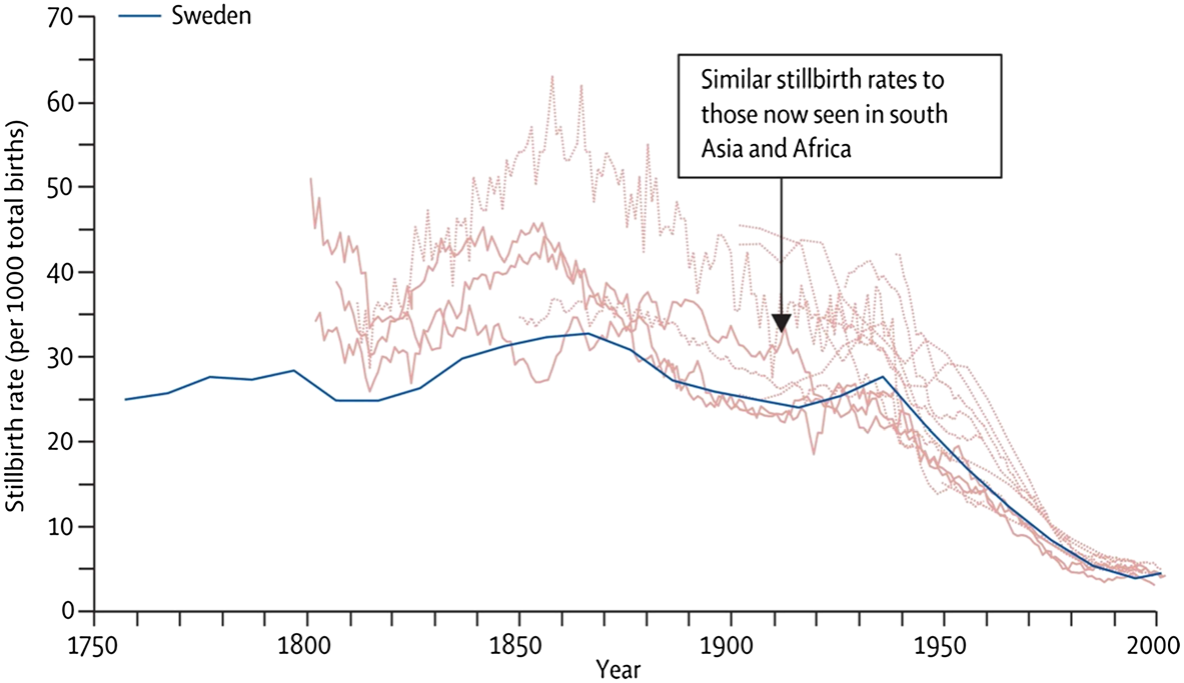
**Historical stillbirth rates for high-income countries.** Reprinted from Goldenberg, 2011 [46].

In HIC, until about 1930, the fetal mortality rates generally ranged from 35 to 50/1000 births. In the recent *Lancet* Stillbirth Series, nearly all HIC reported current stillbirth rates of <5/1000 births with several countries reporting rates as low as 2/1000, representing a reduction of approximately 90% over time [37,38]. As with maternal mortality, the reduction in stillbirth began about 1935, continued relatively rapidly until about 1980, and then has continued more slowly until the present (Figure 3). Since the cause of death for a stillbirth is often less clear than for a maternal death, defining the interventions responsible for the majority of the reduction is more difficult. However, the interventions responsible for some of the reduction in fetal death rates are clear [39-41]. For example, in the 1920’s, syphilis reportedly accounted for up to 20% of US stillbirths. Today in HICs syphilis is rarely a cause a stillbirth. Eighty years ago, in HIC, preeclampsia and eclampsia were the major cause of fetal mortality, but today account for only a small percentage of a much smaller number of stillbirths. Rh disease was until the 1960’s an important cause of stillbirth. With routine Rhogam (anti-D) prophylaxis for Rh negative mothers, and fetal transfusion for severely affected fetuses, stillbirth due to Rh disease is rare. Stillbirths associated with abruption also have been much reduced. Monitoring the fetus for signs of asphyxia prenatally and during delivery, using various techniques such as fetal heart rate monitoring, with delivery for signs of distress, has reduced asphyxia-related fetal mortality. The high rates of cesarean section and labor induction in many HIC occur in part to reduce risk of stillbirth. Thus it appears that with appropriate care for the mother, stillbirth rates have been reduced by up to 90%.

### Fetal mortality: evidence based recommendations

As noted, most stillbirths in LIC are caused by intrauterine asphyxia with a smaller percentage due to infection and an even smaller percentage due to congenital anomalies. Many of the maternal conditions discussed above lead to fetal asphyxia including prolonged labor, preeclampsia/eclampsia and placental abruption. Pregnancy complications such as growth restriction, abnormal presentations, multiple fetuses, and cord complications also kill fetuses through asphyxia. Appropriate obstetric care including monitoring for signs of asphyxia - often with cesarean section for fetal distress - can prevent many stillbirths. Fetal deaths due to infection can in certain cases be prevented. For example, diagnosing and treating maternal syphilis, preventing maternal malaria with bednets and intermittent prophylaxis, and eliminating prolonged labor to reduce asphyxia and bacterial chorioamnionitis, all have a role in reducing stillbirths. With the possible exception of treatment for maternal GBS infection or membrane rupture, maternal antibiotic administration has a smaller role, if any, in preventing stillbirths. Since poor fetal growth is often associated with stillbirth, some studies suggest that maternal nutritional support that increases fetal growth might result in a reduction in stillbirths [28].

#### Neonatal mortality: history

**Figure 4:**
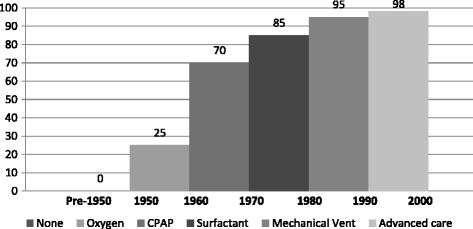
**Estimated reductions in RDS-associated mortality with various interventions, by approximate year of introduction.** Adapted from Kamath B [7].

Neonatal mortality in HIC has also fallen substantially in the last 80 years [42]. In 1935, the neonatal mortality rate was about 35/1000 live births and in recent years the neonatal mortality rate in most HIC has been approximately 3-5/1000 live births. The decline is related both to a decreasing prevalence of some conditions and better treatment for others. About one-third of neonatal mortality is associated with preterm birth. Because in HIC the incidence of prematurity has, if anything, increased over the last three decades, reduction in the prevalence of preterm birth is unlikely to be associated with the decreased mortality [20]. Figure 4 depicts the major advances in care for preterm infants, when they were introduced and the proportion of this decrease likely due to these interventions. Beginning in about 1960, treatment of RDS with oxygen, and later with various types of ventilation support including continuous positive airway pressure (CPAP) and mechanical ventilation, and still later, artificial surfactant, substantially lowered the death rate from RDS [7]. In HICs beginning in the 1990’s, increasing use of maternal corticosteroids prior to delivery for women at risk of preterm delivery substantially lowered the incidence of RDS. The incidence of infection in preterm as well as term newborns was also reduced with increased clean delivery practices and the use of antibiotics [43], saving many lives. Maternal treatment for syphilis and vaccination for tetanus also contributed to the reduction of newborn infection-related mortality [44]. The incidence of newborn asphyxia was substantially reduced with better obstetric care including monitoring for hypoxia prenatally and during labor, and use of cesarean sections for the indication of fetal distress. Newborn resuscitation techniques improved and treatment of asphyxia also reduced mortality [45]. Thus, as seen with maternal and fetal mortality, from about 1935 onward, neonatal mortality rates significantly decreased in HIC.

### Neonatal mortality: evidence based implications

As noted, neonates in LIC die from three major causes; asphyxia, infection, and preterm birth. Asphyxia is best prevented; the same maternal interventions that reduce stillbirth also reduce the prevalence of neonatal asphyxia [46]. Thus, appropriate care for women with obstructed or prolonged labor, abruption, preeclampsia/eclampsia, or for fetal growth restriction will not only reduce stillbirths, but also neonatal asphyxia and neonatal mortality. Neonatal resuscitation is also important in reducing deaths of those infants born with mild asphyxia [46]. Care for newborns with significant asphyxia, including respiratory, temperature and nutritional support, can save some additional lives. Thus, appropriate treatment of maternal conditions as well as newborn resuscitation and neonatal care can have a profound impact on neonatal deaths from asphyxia.

Preventing deaths from neonatal infection also involves multiple approaches, some directed at the mother and some at the neonate. For example, preventing prolonged labor, treating the mother’s syphilis, immunizing her against tetanus and giving antibiotics in the face of maternal colonization with GBS or ruptured membranes will all likely reduce neonatal infection and mortality. The use of sterile/clean techniques including hand-washing at the time of delivery and in the newborn period, prophylactic antibiotics - especially in preterm infants and appropriate cord care – now often with chlorhexidine, especially for home births - also reduce neonatal infections and mortality [47]. For neonatal infections, antibiotic treatment often proves lifesaving. Thus, to achieve substantial reductions in neonatal infection-related mortality, a variety of maternal and newborn interventions are needed.

The predominant cause of preterm neonatal mortality is RDS, but preterm infants also die from other causes including necrotizing enterocolitis, IVH and bacterial infection [20]. While there are few if any interventions that effectively prevent preterm births, prevention of some of the above conditions associated with preterm birth is possible. Depending on the setting, corticosteroids given to the mother in the days prior to delivery may prevent 30% of RDS as well as necrotizing enterocolitis, IVH and neonatal mortality [48]. For those neonates with RDS, oxygen, ventilatory support and surfactant can substantially reduce mortality. Thus, a program for reducing mortality from preterm birth also has maternal and neonatal components, and when they are applied appropriately, much of the preterm neonatal mortality currently occurring in LIC can be eliminated.

### Summary of evidence-based interventions to reduce mortality

The interventions needed to reduce maternal, fetal and neonatal mortality are thus well known and there is every reason to believe that if these were made available within a system of care to pregnant women and their newborns, pregnancy related mortality rates in LIC should approach those in HIC [36]. These types of reductions have been demonstrated in several countries in transition including China, Malaysia and Sri Lanka [1,2,5]. The most important question that arises is not which interventions should be introduced, but how to make these interventions widely available with high quality of care in low and middle income countries. All too often, studies suggest that while an intervention may be available, it is performed on the wrong patients, performed poorly or too late. Thus, not only the coverage of an intervention, but also the quality performance of that intervention is crucial. Finally, it has become clear that introducing one intervention at a time rarely reduces mortality significantly. Instead, developing an understanding of the population that needs to be served and the capabilities within the system to provide care to that population is an appropriate starting point in any geographic area. Often termed “the systems approach” there is substantial evidence that defining the population, the goals of that care, the resources and personnel available in the clinics, hospitals, and in the home, and then creating a system of care - provides the most success in reducing maternal, fetal and neonatal mortality [49]. In summary, to save the life of a mother, fetus or neonate with any particular condition, the condition must either be prevented or be diagnosed and treated in an appropriate and timely manner. Knowing the conditions that kill mothers, fetuses and newborns and when and where they die is therefore crucial.

### General recommendations

**Figure 5:**
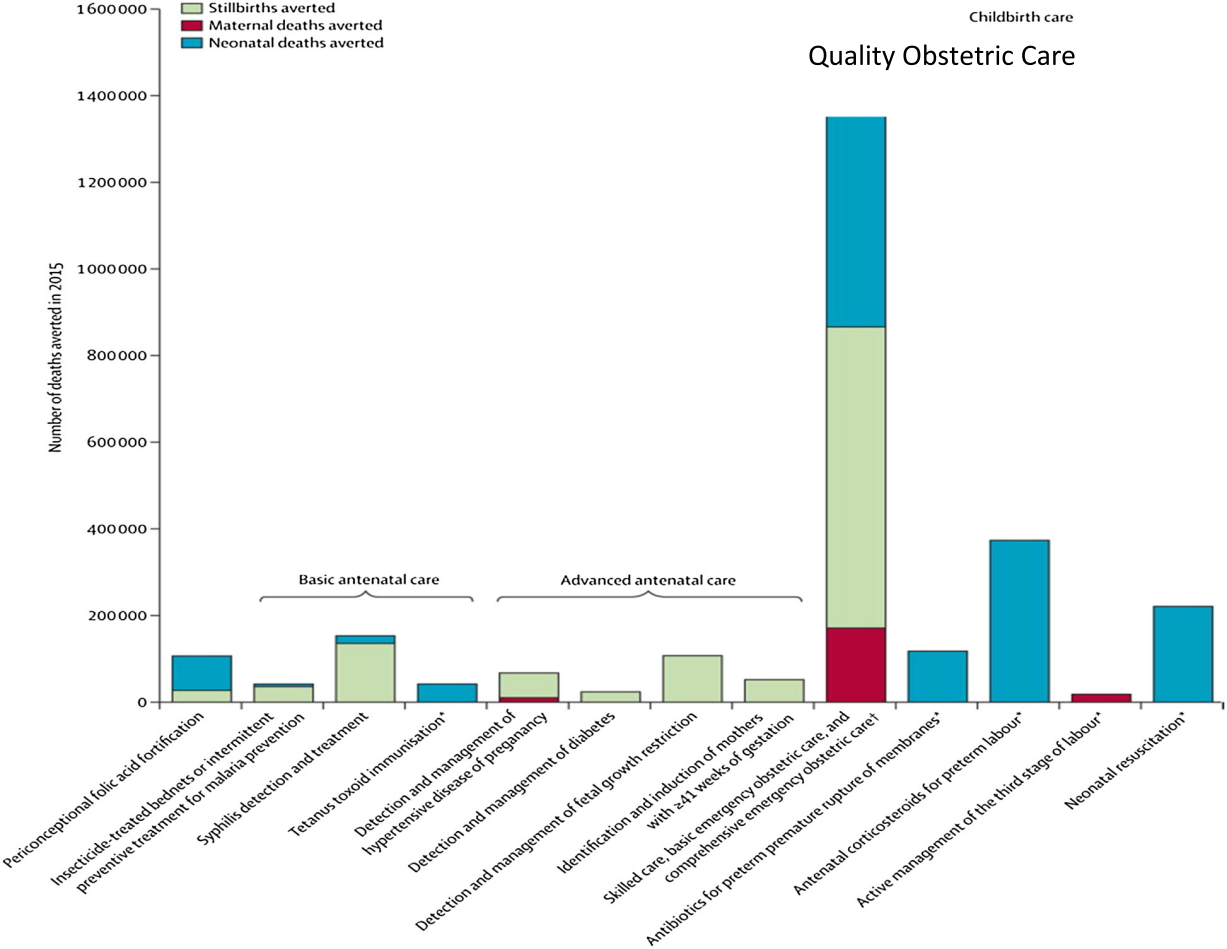
**Potential maternal, fetal and neonatal lives saved by obstetric and newborn interventions.** Adapted from Pattinson R, 2011 [38].

**Figure 6:**
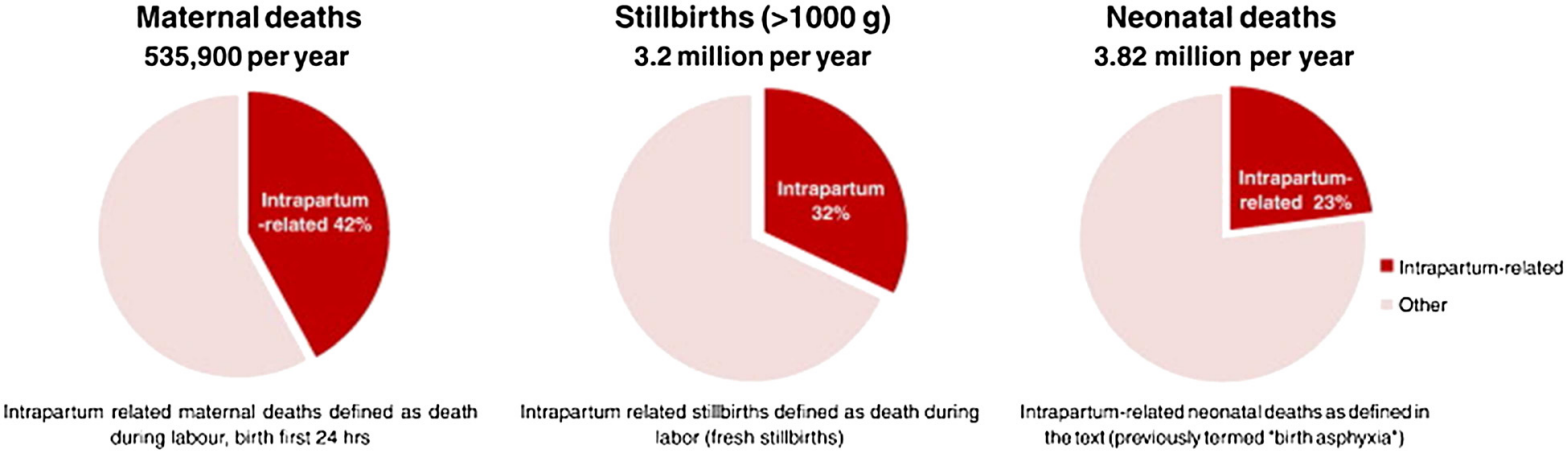
**Intrapartum-related maternal, fetal and neonatal mortality.** Reprinted from Lawn et al. [53].

Based on the information detailed above, proposed lists of interventions appropriate for low and middle-income countries to reduce maternal, fetal, and neonatal mortality, with estimates of potential lives saved, have been proposed [36]. Figure 5, from the *Lancet,* estimates the number of maternal, fetal and neonatal lives saved in low-income countries with various interventions, and emphasizes the importance of basic and emergency care during labor and delivery. Since most maternal and fetal as well as many neonatal deaths occur around the time of delivery as shown in Figure 6 [50], the United Nations has promoted two strategies to reduce mortality: ensuring a skilled birth attendant at delivery and ensuring prompt access to emergency obstetric care. Ideally, all women would have access to essential obstetric care which includes intrapartum monitoring with early detection and management or referral for complications [Table 1]. In this description, basic essential emergency obstetric care is comprised of 6 non-surgical functions including parenteral antibiotics, parenteral oxytocic drugs, parenteral anticonvulsants, manual removal of the placenta, removal of retained products of conception, and assisted vaginal delivery by forceps or vacuum extraction [51]. Neonatal resuscitation is often included in this proposed package. Comprehensive emergency obstetric care would add blood transfusion and cesarean section to the list. Staffing for basic emergency obstetric care would include at least two skilled birth attendants available 24 hours a day, seven days a week assisted by trained staff. Comprehensive emergency obstetric care would require staff trained to provide blood and perform a cesarean section. Since saving many maternal, fetal and newborn lives requires a cesarean section, timely access to this intervention is crucial to achieve mortality rates comparable to those seen in high-income countries.

**Table 1:** **Components of basic and comprehensive emergency obstetric care**

Basic*	**Comprehensive**
Parenteral antibiotics	All components of Basic
Parenteral oxytocic drugs	Blood transfusion
Parenteral anticonvulsants	Cesarean Section
Manual removal of the placenta	
Removal of retained products of conception	
Assisted vaginal delivery by forceps or vacuum	

*Neonatal resuscitation is often included in this proposed package.

### Birth attendant capacity

Within any system of care, the capability of the birth attendant is crucial to reducing maternal, fetal and neonatal mortality. In low-income countries, historically, most often the birth attendant has been an unskilled or traditional birth attendant (TBA) [52]. For the most part, studies have shown that even with additional TBA training, the maternal mortality rates do not decline substantially, although with training in resuscitation, there may be some reduction in stillbirth and neonatal mortality [50,53]. For this reason, the World Health Organization (WHO) and other organizations have recommended the use of skilled birth attendants for delivery. However, the training and skills of these “skilled attendants” vary widely, and many cannot perform a cesarean section, give blood or administer antibiotics, interventions often necessary to save a life [52]. Even the ability of the most skilled attendant to save a life is limited if there is no blood or antibiotics, or if the facilities necessary to do a cesarean section are not available. While a full review is beyond the scope of this manuscript, it should be obvious that in creating an effective system of care, great attention must be paid to the skill level of the birth attendant and the circumstances in which that attendant will attempt to provide life-saving care to the mother, fetus and newborn.

### Data

In low-income countries, one of the major obstacles to program development aimed at improving pregnancy related outcomes is lack of reliable data on these outcomes, the causes of these outcomes and the coverage existing for the interventions that might improve these outcomes. Without these types of data, and the ability for a hospital, a geographic area, or a political district to compare their outcomes with other similar entities, the likelihood that improvement will occur is much reduced [54]. Without the ability for these entities to compare current outcomes to those achieved historically, the impact of newly introduced programs or interventions will not be understood. Only with reliable data used to focus attention on continuous quality of care improvement, is it likely that sustained improvement will occur. In virtually every geographic area where reductions in maternal, fetal and neonatal mortality have occurred, improvements in care and outcomes have gone hand in hand with the development of data systems that capture outcomes, causes of poor outcomes and intervention coverage.

## Conclusions

There is substantial information about the medical interventions that if widely used and used correctly could save many maternal, fetal and neonatal lives. This review also emphasizes that there is no single intervention which will save the majority of lives. Instead, multiple interventions throughout the pregnancy, or even before (family planning) – but especially during delivery, are needed to save lives. Another observation that arises from the review is that the maternal conditions that cause maternal, fetal and neonatal deaths are often the same, as are the treatments to prevent those deaths [Table 2]. Thus, programs focused on reducing all types of pregnancy-related mortality would be more efficient than separate maternal mortality, stillbirth or neonatal mortality reduction programs. Since many of the conditions that kill mothers, fetuses, and newborns cannot be predicted or prevented, ideally all women and newborns should have immediate or rapid access to the interventions that save lives, which for the most part are hospital based. Creating a structure by which these interventions can be appropriately applied to all women and newborns in a timely fashion is the challenge for any low-income country to achieve substantial reductions in maternal, fetal and neonatal mortality [38,40].

**Table 2:** **Major killers of mothers, fetuses and newborns in low-income countries**

**Condition**	**Mother**	**Fetus**	**Newborn**
Hemorrhage	X	X	X
Preeclampsia/eclampsia	X	X	X
Intrauterine infection	X	X	X
Obstructed Labor	X	X	X
Fetal asphyxia		X	X
Preterm labor/birth		X	X
Syphils		X	X
Malaria	X	X	

Adapted from Goldenberg, 2011 [46].

## Abbreviations

CPAP: Continuous positive airway pressure
GBS: Group B streptococcus
HIC: High-income countries
IVH: Intraventricular hemorrhage
LIC: Low-income countries
RDS: Respiratory distress syndrome
TBA: Traditional birth attendant
